# Pathogenesis/genetics of frontotemporal dementia and how it relates to ALS

**DOI:** 10.1016/j.expneurol.2014.06.001

**Published:** 2014-12

**Authors:** Janis Bennion Callister, Stuart M. Pickering-Brown

**Affiliations:** Institute of Brain, Behaviour and Mental Health, University of Manchester, Oxford Road, Manchester, M13 9PT, UK

## Abstract

One of the most interesting findings in the field of neurodegeneration in recent years is tfche discovery of a genetic mutation in the C9orf72 gene, the most common mutation found to be causative of sporadic and familial frontotemporal lobar degeneration (FTLD), amyotrophic lateral sclerosis (ALS) and concomitant FTD-ALS (DeJesus-Hernandez et al., 2011b; Renton et al., 2011). While clinical and molecular data, such as the identification of TDP-43 being a common pathological protein (Neumann et al., 2006) have hinted at such a link for years, the identification of what was formally known as “the chromosome 9 FTLD-ALS gene” has provided a foundation for better understanding of the relationship between the two. Indeed, it is now recognized that ALS and FTLD-TDP represent a disease spectrum. In this review, we will discuss the current genetic and pathological features of the FTLD-ALS spectrum.

## Introduction

Frontotemporal lobar degeneration (FTLD) is a group of complex disorders resulting from the progressive deterioration of the frontal and anterior temporal lobes of the brain. It is the second most common form of presenile dementia (after Alzheimer's disease) with a prevalence estimated between 10 and 30 per 100,000 in individuals between the ages of 45 and 65 years (Sieben et al., 2012). While the grouping of these disorders may give the impression that they have much in common, in fact FTLD is clinically and genetically heterogeneous, and also differs greatly in pathology.

The subcategories of the disease are defined by their dominant clinical symptom in patients; behavioural variant frontotempotal dementia (bvFTD) that accounting for two-thirds of patients, and two language variants classified on their effects on fluency (progressive non-fluent aphasia, PNFA) or semantic difficulties in communicative speech and understanding the semantic content of language (semantic dementia, SD).

Amyotrophic lateral sclerosis (ALS) is a devastating neurodegenerative disease in which the loss of motor neurons from the brain and spinal cord is usually fatal due to respiratory paralysis within 1–5 years of symptom onset (Mitchell and Borasio, 2007). While 1–2 people per 100,000 are currently affected by the disease worldwide, the reality is that 1 in 800 individuals are expected to develop ALS in their lifetime due to the short course of disease progression, making it the most common adult-onset motor neuron disorder (Redler and Dokholyan, 2012).

Prior to the emergence of evidence to the contrary, ALS was widely reported as having a sparing of cognitive ability, sensation, and autonomic nervous function, as a result of the restriction of cell death to the motor neurons. It has not yet been conclusively proven whether the primary site of such dysfunction is the upper motor neurons (UMN) that originate in the motor cortex and are not directly responsible for stimulating the target skeletal muscle, or the lower motor neurons (LMN) that continue the signal to the muscle following glutamate release from the UMN. The interconnectedness of these systems, which are both required for target muscle movement, makes it difficult to decipher the specifics, leading to much debate (Redler and Dokholyan, 2012).

It has now been established that FTLD and ALS can co-occur in the same individual, and more recently the focus has switched away from a simple co-incidence hypothesis to a popular recognition of a spectrum of disease, supported by the clustering of neurodegenerative diseases in relatives of patients with ALS (Al-Chalabi et al., 2012). Up to half of ALS patients show some degree of functional loss in frontal lobe tests, and in 15% of cases this is sufficient to warrant an official diagnosis of FTLD (Ringholz et al., 2005). At the other end of the spectrum, around 40% of FTLD cases have measurable motor dysfunction with up to 15% fitting with the ALS classification (Burrell et al., 2011).

With the emergence of this relationship between FTLD and ALS, it has become more important to use care when referring to any given point on the spectrum, and so nomenclature has naturally developed alongside it. The current terminology refers to patients who do not meet the criteria for FTLD, but do have behavioural or cognitive deficits as ALS with cognitive or behavioural impairment (ALS Ci/ALS Bi). Patients with FTLD who show some motor neuron involvement on a clinical or electronmyograph level without actually developing ALS are referred to as FTLD-MND or FTLD-MND-like. Those who fall at the midway point are referred to as ALS-FTLD or FTLD-ALS; the order is usually dependent on the clinical symptoms that appeared first. Now that the spectrum is widely accepted within the FTLD and ALS research community, it makes sense that novel findings in either field will be investigated in the other, and a clearer picture will start to emerge.

## ‘Pure’ FTLD

### MAPT

FTLD is a proteinopathy that can be sub-categorized pathologically based on the major constituent of the abnormal, ubiquitinated protein inclusions that characteristically reside in the cytoplasm and nucleus of neuronal and glial cells.

The first reported genetic linkage in FTLD families with autosomal dominant disinhibition, dementia, parkinsonism, and amyotrophy was to chromosome 17q21 (Wilhelmsen et al., 1994), and was subsequently, following a consensus conference, named FTDP-17 (Foster et al., 1997, Hutton et al., 1998, Poorkaj et al., 1998, Spillantini et al., 1998b). The knowledge that most FTDP-17 cases presented with inclusions that stained positive for the microtubule-associated protein Tau (albeit with great variability) led to analysis of the *MAPT* gene on chromosome 17q21. These investigations identified the first novel missense and slice-site mutations in *MAPT* associated with FTLD (Clark et al., 1998, Hutton et al., 1998, Spillantini et al., 1998a).

Following on from those initial mutations, a total of 44 different *MAPT* mutations have been reported (http://www.molgen.ua.ac.be/). These changes result in either an exonic missense mutation or interference with alternative splicing, disrupting the ratio of tau isoform expression (Hutton et al., 1998).

The normal function of Tau is to promote the assembly of tubulin microtubules via interaction with its microtubule domain, modulating stability (Lee et al., 1989). It is a phosphoprotein mainly expressed in neurons, with phosphorylation status important for microtubule binding (Biernat et al., 1993, Gustke et al., 1992, Miyasaka et al., 1993). There are six tau isoforms that are expressed in adult brain tissue, produced by alternative splicing of exons 2, 3, and 10 (Goedert et al., 1989). Half the naturally occurring isoforms of tau contain three imperfect repeats of ~ 32 amino acids in the microtubule domain at the c-terminus (3R) and the other contain four (4R), controlled by alternative splicing of exon 10. The 4R form of tau has the strongest association with microtubules of the two and is often referred to as being more ‘sticky’ (Gustke et al., 1994). Mutations that disturb alternative splicing regulation lead to an increase in the 4R form over 3R, and (along with missense mutations in exon 10) are associated with a tauopathy composed of four-repeat tau.

Families with missense or splice site mutations affecting exon 10 have neuronal and glial inclusions while families with mutations outside of exon 10 have neuronal inclusions only, comprised of all six isoforms (Grover et al., 1999). Disruption of a normal equimolar 3R to 4R ratio of tau isoforms may be sufficient to drive aggregation, as it has been shown that even spiking small amounts of 3R tau into a 4R aggregation model inhibits assembly (Hutton, 2001).

### Progranulin

Following the discovery of *MAPT* mutations in FTDP-17 families, evidence started to amass that it may not be the sole gene responsible for the disease in patients with linkage to this region. Those showing clinical symptoms of FTD underwent extensive mutation analysis and some were found to have mutation-free *MAPT*. Further investigation of neuronal inclusions did not show Tau-positive staining, but did stain for ubiquitin (Rosso and van Swieten, 2002). In 2006, mutational analysis of nearby genes showed that indeed there was a second gene, *granulin* (*GRN*), within the region of chromosome17q21, 1.7 Mb centromeric to *MAPT*, which had mutations (Baker et al., 2006, Cruts et al., 2006).

This *GRN* gene encodes a cysteine-rich secreted glycoprotein (Songsrirote et al., 2010) implicated in tissue repair (He and Bateman, 2003), glucose sensing (Kim et al., 2011), and cancer (He and Bateman, 1999, He et al., 2002, Swamydas et al., 2011). The gene product of *GRN*, progranulin (PGRN) can be proteolytically cleaved by enzymes such as elastase into small peptides, known as granulins (Zhu et al., 2002). Granulins arise from a 12 cysteine granulin motif that form (pre-cleavage) six disulphide bridges (Hrabal et al., 1996) resulting in a parallel stack of beta-hairpins (Hrabal et al., 1996, Tolkatchev et al., 2008). Full-length progranulin also contains a signal peptide, which aids in transportation to the Golgi and through to the plasma membrane for secretion. During progress through the endoplasmic reticulum, carbohydrates are added at several aspargine-linked glycosylation sequences and it is finally targeted to the plasma membrane where the signal peptide is cleaved and the mature glycoprotein secreted.

Until late 2011 mutations in the *GRN* gene were the most frequent known cause of familial FTLD, at up to 20% (Baker et al., 2006, Cruts et al., 2006, Mackenzie et al., 2010, Rademakers and Hutton, 2007). Pathological *GRN* mutations found to date cause disease as a function of haploinsufficiency, with mutations producing a premature termination of the coding sequence being largely to blame. The resultant messenger RNA (mRNA) undergoes nonsense-mediated decay and the truncated protein is not translated. In addition, a splicing mutation leading to retention of an intron and nuclear degradation of GRN mRNA can also be a cause (Cruts et al., 2006).

There are a number of non-synonymous substitutions known for PGRN (see database at http://www.molgen.ua.ac.be/ADMutations/), though to date only one of these has been confirmed as truly pathogenic. This change of an alanine to an aspartic acid (A9D) within the signal peptide at the N-terminus of PGRN results in a loss of PGRN secretion (Mukherjee et al., 2006).

Screening of ALS and ALS-FTD patients shows that GRN mutations are not a common cause of ALS phenotypes, placing PGRN at the FTLD end of the spectrum (Schymick et al., 2007). There has been a first report of a patient with an A9D mutation with clinical and pathological symptoms of ALS (Cannon et al., 2013). It should be noted that this is currently confined to a single case, and as such it is possible that the patient had concomitant ALS. More research in this area is required.

Upon autopsy, individuals with FTLD with *GRN* mutations exhibit cerebral atrophy (most severe in the frontal lobes), frequently with a shrunken caudate nucleus and a loss of substantia nigra pigmentation (Mackenzie et al., 2006b). The majority of cases have also suffered a loss of pyramidal neurons. While FTLD-TDP molecular pathology is heterogeneous, the findings in cases with PGRN mutation show a highly consistent pattern corresponding to FTLD-TDP Type A (see below), that being numerous short dystrophic neurites (DN) and crescentic or oval neuronal cytoplasmic inclusions (NCI), concentrated primarily in neocortical layer 2. In addition, this subtype is also has a moderate number of lentiform neuronal intranuclear inclusions (NII) (Mackenzie et al., 2011).

## The middle ground

### TDP-43

TAR DNA-binding protein (TDP-43) is a 414 amino acid nuclear protein, encoded by *TARDBP*, a chromosome 1 gene. Before its role in FTLD and ALS was uncovered, TARDBP was cloned from a screen for TAR DNA of HIV type 1 binding, and found to be a heterogeneous ribonucleoprotein (hnRNP) with two RNA recognition motifs (Kumar-Singh, 2011). Functioning as a transcriptional regulator involved in RNA splicing and stability. TDP-43 has since been identified as a component of NCI in all FTLD-TDP43 subtypes, as well as in sporadic ALS (sALS) (Neumann et al., 2006). While missense and nonsense mutations in TARDBP do occur on the FTLD-ALS spectrum, they do so with a frequency of less than 1% in FTLD and around 1% in ALS (Van Langenhove et al., 2012).

In 2006, the majority of cases with tau-negative inclusions that stained positive for ubiquitin in FTLD (known as FTLD-U) were found to contain TDP-43 protein, as did the majority of sALS and some fALS cases (Neumann et al., 2006). This information, combined with work by the same group categorising subtypes of FTLD-U by differential labeling of pathology (Sampathu et al., 2006) and a second group studying clinicopathological correlations (Mackenzie et al., 2006a) a clear pattern started to emerge, followed by combined classification system for what are now known as the FTLD-TDP subtypes (Mackenzie et al., 2011).

Around half of FTLD-TDP type A are familial cases, and as already mentioned earlier, are most often associated with progranulin mutations (which are always FTLD-TDP43 type A). More recently they have been found also to contain the repeat expansion mutation in the C9orf72 gene (described below). The inclusions found in this subtype include neuronal cytoplasmic inclusions (NCIs) neuronal intranuclear inclusions (NIIs) and dystrophic neurites (DNs), usually in layer II of the cerebral cortex. Clinically, the presentation is usually bvFTD or PNFA (and occasionally with SD).

Type B FTLD-TDP43 is associated with FTD-ALS and bvFTD. Pathology also includes NCIs, though fewer and they can be throughout the entire cortical thickness, while NIIs and DNs are rare. Males are more frequently affected in this category, and as might be expected from the ALS link, it is the group with the shortest life expectancy at just over five years on average. Early studies genetically linked many cases of this subtype to chromosome 9p, now known to be caused by a hexanucleotide expansion mutation (GGGGCC) in the gene *C9orf72*.

Type C FTLD-TDP43, like type A, is predominantly in layer II and is most frequently associated with SD (and occasionally bvFTD). It features long DN in superficial cortical layers, and no current linkage to any gene.

Type D FTLD-TDP43 have few NCI, and instead many NII and DN throughout all layers. This subtype is associated with valosin-containing protein (*VCP)* mutations, and is very rare at less than 1% of familial FTLD. Clinical presentation for type D is familial Inclusion body myopathy with Paget Disease of Bone and frontotemporal dementia (IBMPFD).

TDP-43 is predominantly found in the nucleus, but shuttles between there and the cytoplasm, where it is present only at low levels. When this translocation to the nucleus is inhibited in cells in culture, TDP-43 is accumulated and sequestered as cytoplasmic aggregates, echoing pathological findings in brain and spinal cord sections from FTLD-U and sALS cases showing clearance of nuclear TDP43 in favour of similar cytoplasmic aggregates (Winton et al., 2008).

The neuropathology of sALS is in most cases characterized by TDP-43 cytoplasmic accumulation in neurons and glia of the primary motor cortex, brainstem motor nuclei, spinal cord, and associated white matter tracts(Mackenzie et al., 2007). Such cases are termed ALS-TDP, and this pathology does not overlap with *SOD1* mutations, which are discussed below. However TDP-43 was identified from a genome wide RNAi screen for SOD1 regulators, an observation confirmed by biochemical analysis that provides an interesting link between SOD1 fALS and ALS-TDP (Somalinga et al., 2012).

### FUS

Fused in sarcoma (FUS, also known as translocated in liposarcoma, TLS) is a 526 amino acid protein with several conserved domains; a transcriptional activation domain, multiple nucleic acid binding domains (three arginine-glycine-glycine boxes, an RNA recognition motif, a zinc-finger) and a nuclear localization signal (NLS). It was identified as a fusion oncogene causing human myxoid liposarcomas, where aberrant chromosomal translocation results in the N-terminus being fused to a transcription factor (Dormann and Haass, 2013). When in the nucleus, FUS is thought to be involved in regulation of transcription and pre-mRNA splicing. Cytoplasmic FUS in neurons appears to have a role in mRNA transport, where it can potentially facilitate local protein synthesis at synapse (Colombrita et al., 2012).

Mutations in the *FUS* gene were identified as a cause of fALS in 2009, representing around 4% of fALS in the studies with 14 mutations in 26 unrelated families (Kwiatkowski et al., 2009, Vance et al., 2009). The FUS protein was found to be deposited in cytoplasmic inclusions in these patients (Kwiatkowski et al., 2009). Most mutations were missense, autosomal dominant and affected exon 15, which encodes the c-terminus of the protein. This clustering of mutations around exon 15 disrupts binding of FUS to Transportin, a nuclear import receptor that shuttles proteins with a non-canonical NLS (such as that of FUS) from the cytoplasm to the nucleus (Lee et al., 2006). This disruption by missense mutation in turn leads to an accumulation of mutant FUS in the cytoplasm (see (Dormann and Haass, 2013) for a full review). FUS pathology is often associated with a reduction in nuclear FUS staining consistent with the idea that a nuclear importation defect is responsible (Dormann and Haass, 2011). Interestingly, in cultured human cells a fraction of TDP-43 is shown to be complexed with FUS, and this interaction is enhanced by TDP-43 mutant expression(Kim et al., 2010).

De novo mutations of *FUS* also account for a portion of sporadic ALS cases. A splice-site mutation in FUS intron 13 leads to C-terminal truncation of the protein (IVS13-2A > G) (DeJesus-Hernandez et al., 2010), and a point mutation in exon fifteen leads to missense mutation R521C(Chio et al., 2011), a mutation that also occurs in fALS.

Though no patients from the above studies had features of FTLD, studies by Neumann et al. (Neumann et al., 2009) found FUS co-localized with all the ubiquitin-immunoreactive (ub-ir) inclusions in their atypical FTLD-U (aFTLD-U) cases including DN, NCI and NII, along with additional inclusions in glial cells that were not ub-ir. This pathology was observed in the absence of *FUS* mutation and this subtype is now known as FTLD-FUS.

### VCP

Valosin-containing protein (VCP, also known as p97) is a conserved, multifunctional protein essential for growth in mice and other model organisms (Muller et al., 2007). Highly abundant, it comprises around 1% of total cellular protein and is a member of the class II AAA (ATPases associated with diverse cellular activities) family. VCP forms homohexamers, and N-terminal domain (N-domain) binding of various cofactors plays a role in the multi-functionality of the protein, enabling it to target specific substrates for degradation via the ubiquitin–proteasome system. Known roles include ER and golgi reassembly, nuclear envelope regeneration, proteolysis, spindle disassembly, chromosome condensation, DNA damage response, DNA replication, suppression of protein aggregation, autophagy, ER-associated protein degradation and sex determination (Yamanaka et al., 2012).

More recent research has enhanced our understanding of VCP's mode of action with relevance to neurodegenerative disease. VCP protein contains two ATPase domains (known as D1 and D2), and its deficiency results in mitochondrial uncoupling and a significant reduction of cellular ATP production (Bartolome et al., 2013). This decrease in ATP levels lowers the energy capacity of the cell, rendering them more vulnerable to high energy-demanding processes such as ischemia. Research has also shown that stress granule clearance, a process important for clearance of pathogenic ribonucleoprotein is impaired by depletion or pathogenic mutation of VCP (Buchan et al., 2013).

Ubiquitinated inclusions found in muscle, bone and brain are a feature of inclusion body myopathy with Paget's disease of bone and frontotemporal dementia (IBMPFD), which can lead to disabling muscle weakness (Inclusion body myopathy-IBM), osteolytic bone lesions (Paget's disease of bone-PDB), and neurodegeneration (FTD). *VCP* mutations are an underlying cause of IBMPFD, and 6 missense mutations were found in a total of 13 families with the disease originally (Watts et al., 2004), with the number of identified mutations now around 20 from 50 unrelated families (http://www.molgen.ua.ac.be/ftdmutations).

Myopathy is the most common clinical symptom of individuals with IBMPFD occurring in 80–90% of patients and manifesting as adult-onset (~ 44 years) with proximal and distal muscle weakness (Guinto et al., 2007). Patients show a progressive muscle weakness typically starting in pelvic and shoulder region and spreading to the heart and respiratory system. Though this may be confused with ALS due to the age of onset, muscle weakness, and even evidence of neuropathic changes usually consistent with motor neuron degeneration, electromyography shows myopathic changes in affected individuals, and histological analysis of affected muscles by biopsy shows myonuclear and sarcoplasmic inclusion bodies reactive with ubiquitin and TDP-43 (Nalbandian et al., 2011). The dementia aspect of the disease presents later (~ 54 years old), and is only present in around a third of patients.

In terms of neurodegeneration, *VCP* was considered an FTLD-related gene, due to its characteristic language and/or behavioural dysfunction and FTLD-TDP D type pathology. However, mutations have also been found to be responsible for autosomal dominant fALS in an Italian family in which *SOD1*, *TARDBP* and *FUS* mutations were previously excluded (Johnson et al., 2010). This finding demonstrates that some individuals with a VCP mutation do have motor neuron disease as part of the phenotype along with myopathy, further muddying the water in diagnosis of disease. Further analysis has shown that *VCP* mutations were present in ~ 1–2% of a large cohort of fALS cases from unrelated families, and other research has supported this interpretation (DeJesus-Hernandez et al., 2011a, Shaw, 2010).

The most common mutation of *VCP* in FTLD or ALS is R155H, which like other *VCP* mutant gene products results in a normal hexametric structure (Weihl et al., 2006). However, mutations here induce conformational alterations in the N-domain, resulting from impaired communication between the D1 and N domains (Fernandez-Saiz and Buchberger, 2010). It has been suggested that this imbalanced co-factor binding is an important determinant of IBMPFD pathology, possibly by trapping functioning VCP in unproductive co-factor complexes. The sheer number of co-factor interactions with VCP may underlie the highly variable nature of pathology and clinical presentation.

### C9orf72

The *C9orf72* gene encodes a protein of unknown function, recently identified in silico as a potential DENN-type GEF (Levine et al., 2013, Zhang et al., 2012). DENN (differentially expressed in normal and neoplastic cells) domain proteins act as specific regulators of the Rab GTPase family, functioning enzymatically as guanine nucleotide exchange factors (GEFs). A likely function of the gene product based on structural similarity, is to regulate membrane traffic in conjunction with Rab-GTPase switches, though this has yet to be validated experimentally (Levine et al., 2013).

In late 2011, a repeat mutation in the first intron of *C9orf72* was found to be the most common genetic cause of cause of fALS and FTD (DeJesus-Hernandez et al., 2011b, Renton et al., 2011). This mutation is thought to be responsible for around 40% of fALS and 21% of FTD., segregating perfectly with disease. The mutation is a hexanucleotide repeat with the sequence GGGGCC, ranging from zero-30 copies in unaffected individuals, to an excess of four thousand in mutation carriers, and reduces the expression of at least one RNA species (DeJesus-Hernandez et al., 2011b, Gijselinck et al., 2012, Renton et al., 2011), and forms nuclear RNA foci (DeJesus-Hernandez et al., 2011b).

This C9orf72 mutation is also the most frequent cause of apparently sporadic ALS and FTD found to date, accounting for 5–7% of cases in white Americans, Europeans and Australians (Majounie et al., 2012). Majounie *et al*. postulate that these cases are actually cryptically related familial ones, occurring for various reasons such as unfamiliarity with the pedigree, previous generations dying at a young age before onset of neurological symptoms and incomplete penetrance of the mutation. Though the mechanism behind it is unclear, the finding that penetrance of this mutation seems to be complete only at a late stage of life argues against the notion that late-onset neurodegeneration has non-genetic etiologies.

This mutation makes *C9orf72* cases of FTLD/ALS the most recent addition to the DNA-repeat expansion associated disease. More than 40 other neurological, neurodegenerative or neuromuscular disorders are linked to repeat instability, which unlike static mutations, have a dynamic repeat process with products continuing to mutate across generations and in different tissue types.

The appearance of *C9orf72* mutations is frequently histologically categorized as a type B pathology, with inclusion bodies in neurons and in glial cells (GCI) that are TDP-43 positive. Other expansion carriers, however, have a type A pathology with DN and NCI in the outer layers of the cerebral cortex (Liu et al., 2013, Snowden et al., 2012).

Although the hexanucleotide repeat is found in the non-coding region of the *C9orf72* gene, an interesting phenomenon known as repeat-associated non-ATG dependent translation (RAN translation) allows expression of mutant proteins made of dipeptide repeats (DPRs) from the expansion (Ash et al., 2013, Mori et al., 2013). RAN translation occurs in the absence of the ATG codon and from both strands. Usually required to initiate this form of translation is long hairpin forming repeats as first described in relation to spinocerebellar ataxia type 8 and myotonic dystrophy type 1 (Zu et al., 2011). Antibodies generated against C9orf72 repeat DPRs from the forward strand of the mutation detect high molecular weight material in brain homogenate from western blotting, and detects neuronal inclusions through the central nervous system of C9 FTLD/ALS cases (Ash et al., 2013). The alternative frame translation products produced by the forward strand are (Glycine-Arginine)_n_ (Glycine-Proline)_n_ (Glycine-Alanine)_n_, and as there is no stop codon present in the (GGGGCC)_n_ repeat, once RAN translation is initiated it may produce extremely large proteins, repeat-size dependent. DPRs from the alternate strand are also found within the cytoplasmic and intra nuclear inclusions in cases with the expansion (Mann et al., 2013).

Though aberrant proteins may be the cause of some of the pathological effects of the C9orf72 expansion mutation, there is not currently sufficient evidence to state that they are the sole cause. Other research points towards a ‘toxic RNA’ hypothesis (Haeusler et al., 2014, Lee et al., 2013) and current thinking in the field often refers to the possibility of a multiple toxic effect of both the mutant protein and RNA. Studies have shown that the RNA of the C9orf72 expansion has a propensity for forming highly stable guanine quadruplexes (G-quadruplexes), and secondary structures formed from short tracts of G-rich sequence associating together (Fratta et al., 2012). RNA foci composed of the hexanucleotide repeat have been found in brain tissue, but their role in pathogenicity is as yet unclear (van Blitterswijk et al., 2012).

### P62/sequestosome-1

p62, encoded for by the *SQSTM1* gene, is another multifunctional protein at the intersection of ALS and FTLD pathology. Unlike the other genes mentioned in this review, its inclusion in the spectrum was found through a candidate gene approach following the observation of involvement of the p62 in ALS (Fecto et al., 2011). p62 is a stress-inducible intracellular protein, and is involved in the regulation of cell survival and death via regulation of cell signal transduction. As is common for such pathways, a mechanism of feedback occurs in that p62 can both suppress autophagy via activation of the mammalian target of rapamycin complex 1 (TORC1) and can itself be regulated by autophagy in terms of protein levels (Komatsu et al., 2012). An N-terminal Phox and Bam1p (PB1) domain is responsible for self- and hetero oligomerization of p62, and targeting of the protein to the autophagosome is also dependent on this region of the protein (Itakura and Mizushima, 2011). Here it interacts with LC3, is incorporated into the autophagosome and is degraded by autophagy.

Inclusions positive for p62 can be found in C9orf72 expansion mutation patients, both with and without TDP-43 pathology. These latter inclusions stain for p62 and ubiquitin only, and are frequently reported as presented with compact globular or star-shaped NCIs and spherical NIIs, abundant in the granular layer (Liu et al., 2013). These NCI and GCI found in the frontal neocortex, cerebellum and hippocampus are rare in non- C9orf72 cases, and so are regarded as the pathological hallmark of C9orf72 mutation carriers.

At its c-terminal, p62 has a ubiquitin-associated (UBA) domain, and is a marker for ubiquitinated cargos targeted for proteosomal degradation. Originally, mutations in the section of *SQSTM1* encoding the UBA domain were reported to cause Paget's disease of bone (PDB) (Goode and Layfield, 2010), highlighting a relationship to IBMPFD. Following the candidate gene approach identification of *SQSTM1* mutation, it has been further reported in ALS and in FTLD (Chen et al., 2014, Hirano et al., 2013, Le Ber et al., 2013, Rubino et al., 2012, Shimizu et al., 2013, Teyssou et al., 2013).

### Ubiquilin 2

The *UBQLN2* gene encodes ubiquilin-2 (UBQLN2), a member of the ubiquilin (UBQLN) family that regulates degradation of ubiquitinated proteins. Mutations in this gene have been found in very rare cases of dominantly inherited chromosome-X-linked ALS (X-ALS) and ALS with FTLD (Deng et al., 2011). Pathological analysis of these individuals revealed axonal loss in the cortico-spinal tract, loss of anterior horn cells, and astrocytosis in the anterior horn of the spinal cord. UBQLN2 positive inclusions were detected in spinal motor neurons of mutation carriers, along with immunoreactivity with p62, ubiquitin, and FUS but not SOD1. UBQLN2 and ubiquitin also co-localize in inclusions of the hippocampus in brain tissue from dementia-linked *UBQLN2* mutation.

Interestingly, UBQLN2 pathology could also be found in brain and spinal cord tissue of ALS or FTLD patients without mutated a *UBQLN2* gene (Deng et al., 2011). This UBQLN (ubiquilin family rather than UBQLN2 specifically) pathology in ALS and FTLD-TDP cases with a C9ORF72 expansion was confirmed by others and is highly distinct (Brettschneider et al., 2012). In the hippocampus of FTLD-TDP and ALS with C9ORF72 expansion, dystrophic neurites that showed focal swellings and dot-like stipples and irregular, aggregate-like formations were extensive in the hippocampal molecular layer and in the CA1–CA4 region. The clearly distinguishable neuropathological disease signature observed by the authors in ALS and FTLD-TDP cases with and without C9ORF72 expansion, suggests a pathophysiological link between C9ORF72 and UBQLN pathology.

## ‘Pure’ ALS

### SOD1

Superoxide dismutase 1 (*SOD1*) was the first gene identified to cause familial ALS (FALS) in 1993, with the original authors citing 11 different missense mutations in 13 families (Rosen et al., 1993). Since then, more than 170 mutations have been recorded in positions that span the whole of the resulting protein (http://alsod.iop.kcl.ac.uk/Als/index.aspx).12% of fALS, while absent in ALS-FTLD (Chio et al., 2012), have not been found in FTLD patients to date. Individuals with *SOD1* mutations have ubiquitin-positive neuronal inclusions, but they are negative for TDP-43 immunoreactivity (Mackenzie et al., 2007).

SOD1 is a 153 amino acid protein predominantly expressed in the cytosol of most cells that are exposed to oxygen. It forms a homodimer following zinc and copper ion complexing that acts as a dismutase; removing superoxide radicals by metabolizing them to molecular oxygen and hydrogen peroxide, thus providing a defense against oxygen toxicity. Mice lacking SOD1 suffer extensive oxidative damage in the cytoplasm of liver cells from as early as 3 months, leading to persistent and widespread damage and hepatocarcinogenesis in later life (Elchuri et al., 2005).

The majority of ALS *SOD1* mutations are of the missense variety, with a few C-terminal truncations due to nonsense or deletion mutations. The mutations are mostly dominant with the notable exception of D90A, recessive in Scandinavian populations for reasons as yet not fully understood (Robberecht and Philips, 2013). In terms of stability, some mutations (for example, C6S and D90A) are as stable as the native SOD1 molecule, and others (for example A4V and G127del) are highly unstable. Most mutations have been found to cause a reduction in dismutase activity, while some (for example G37R, A89V and D90A) are essentially normal or only slightly reduced in activity (Andersen and Al-Chalabi, 2011).

The debate over loss or gain of function that has revolved around these mutations has now been quashed with the use of mouse models. Transgenic mice over-expressing mutant human SOD1 have increased SOD1 activity and a loss of motor neurons that models human ALS, and mice carrying a mutant SOD1 transgene (tgSOD1G85R) on a normal mouse background compared with the same transgene in a SOD1 null background showed no change in survival. (Bruijn et al., 1998). While this ruled out the role of loss of function of SOD1, it has been suggested that while not causative, there may be a modifying effect of loss of SOD1 function in ALS (Saccon et al., 2013).

The most common underlying mutation in *SOD1* varies with geographical location. In the U.S.A., it is A4V, with around 50% of SOD1-ALS patients carrying this mutation. In Japan it is H46R (in the catalytic copper ion binding site), affecting 40% of SOD1-ALS patients with a much longer disease course than the average (around 15 years).

So what is the cause of motor neuron death associated with mutant *SOD1*, if not a change in stability or function? The finding that the SOD1 protein was actually present in aggregates in fALS postmortem patients and also transgenic mice suggested that, similar to other neurodegenerative diseases, the aggregating pathology of ALS could be linked to mutations in this gene (Bruijn et al., 1998). Wild type SOD1 lacking both its metal ions gives rise to soluble oligomers under aerobic physiological conditions, formed by intermolecular disulphide covalent bonds and non-covalent interactions between beta-strands (Banci et al., 2007). The overarching theme then, borne out by further experiments on known fALS mutations, is that metal-free SOD1 is a cause of ALS, and that some mutants associated with the disease may be more prone to oligomerize in vivo due to alterations in metal binding or the stability of that binding (Banci et al., 2008).

## Concluding remarks

We have gained much in our understanding of FTLD and ALS in recent years. From the progress that has been made in our understanding of the molecular genetics of FTLD and ALS over this time, it is clear that at least a subsection of these disorders form part of a disease spectrum with the same gene implicated in both. However, it is clear that there are subtypes which appear, generally, to be exclusive. The reports of ALS in cases with MAPT and PGRN mutations have to be confirmed as does dementia in ALS resulting from SOD1 mutations. Nevertheless, one observation that has come from the realization of a disease spectrum is that it is highly likely that any drugs that are developed for one end will likely be efficacious for the other.
